# True bugs (Hemiptera, Heteroptera) as psyllid predators (Hemiptera, Psylloidea)

**DOI:** 10.3897/zookeys.319.4316

**Published:** 2013-07-30

**Authors:** Dušanka Jerinić-Prodanović, Ljiljana Protić

**Affiliations:** 1 University of Belgrade, Faculty of Agriculture, Nemanjina 6, 11080 Zemun, Serbia; 2 Natural History Museum, Njegoševa 51, 11000 Belgrade, Serbia

**Keywords:** Psylloidea, Heteroptera, predators, natural enemies, Serbia

## Abstract

Data on natural enemies of psyllids are rare and can usually be found in papers about economically significant species. During an investigation of psyllid fauna in Serbia, natural enemies were investigated, too. True bugs were the most numerous among them. From 28 psyllid species, 21 species of true bugs from families Anthocoridae and Miridae were reared. Seven species of Anthocoridae were identified: *Anthocoris amplicollis* (Horváth, 1839), *Anthocoris confusus* Reuter, 1884, *Anthocoris nemoralis* (Fabricius, 1794), *Anthocoris nemorum* (Linnaeus, 1761), *Orius majusculus* Reuter, 1884, *Orius minutus* (Linnaeus, 1758) and *Orius niger* Wolff, 1811. The following 14 species of Miridae were identified: *Atractotomus mali* Meyer-Dür, 1843, *Campylomma verbasci* (Meyer-Dür, 1843), *Deraeocoris flavilinea* (A. Costa, 1862), *Deraeocoris ruber* (Linnaeus, 1758), *Deraeocoris lutescens* (Schilling, 1836), *Heterocordylus genistae* (Scopoli, 1763), *Hypseloecus visci* (Puton, 1888), *Malacocoris chlorizans* Panzer, 1794, *Miris striatus* (Linnaeus, 1758), *Orthotylus marginalis* Reuter, 1884, *Psallus assimilis* Stichel, 1956, *Psallus quercus* Kirschbaum, 1856, *Psallus flavellus* Stichel, 1933 and *Pseudoloxops coccinea* (Meyer-Dür, 1843). The aim of the research was to provide list of true bugs recorded as predators of psyllids in order to preserve their diversity and significance, especially on cultivated plants.

## Introduction

Predators of psyllids (Psylloidea) have been poorly known. So far, detailed researches were carried out only on the predators of economically significant species, such as pear psyllids *Cacopsylla pyri* (Linnaeus, 1758), *Cacopsylla pyricola* (Foerster, 1848) and *Cacopsylla pyrisuga* (Foerster, 1848); apple psyllid *Cacopsylla mali* (Schmidberger, 1836) and eucalyptus psyllids from the subfamily Spondyliaspidinae (Jonsson 1983, Herard 1985, 1986, Santas 1987, Erler 2004, Horton et al. 2004, Sigsgaard et al. 2006, Jauset et al. 2006, Luiz de Queiroz et al. 2012). There are too few data on predators of other psyllid species. Hodkinson and Flint (1971) investigated predators of ash psyllid, *Psyllopsis fraxini* (Linnaeus, 1758), in England, and Harizanova et al. (2012), predatory complex of *Acizzia jamatonica* (Kuwayama, 1908) in Bulgaria. In these papers, the most represented are psyllid predators from the order Hemiptera (suborder Heteroptera) followed by Coleoptera, Neuroptera, Diptera, Dermaptera and Acari. Within the Heteroptera, the most numerous in species families are Anthocoridae, Miridae and Nabidae. A polyphagous species, *Anthocoris nemoralis* (Fabricius, 1794), was most frequently found, with a preference for the species from superfamily Psylloidea (Jonsson 1983, Herard 1986). *Anthocoris nemoralis* (Anthocoridae) was introduced from Europe to North America (British Columbia) in 1963 in order to control *Cacopsylla pyricola*, where its establishment was successful. Besides giving satisfactory effects, this species also spread in the new environment suppressing autochthonous species *Anthocoris antevolens* White, 1879 and *Anthocoris melanocerus* Reuter, 1884, which are most common anthocorid predators in orchards (Herard 1986, Horton et al. 2004).

Data on psyllid predators in Serbia relate only to the predators of pear psyllids (Pavićević 1977, Grbić et al. 1989, Jerinić-Prodanović et al. 2010).

Pavićević (1977) found a large number of predatory species, among which two were from family Anthocoridae. Grbić et al. (1989) recorded four species of Heteroptera: *Anthocoris nemoralis* and *Orius* sp. (both Anthocoridae), *Pilophorus clavatus* (Linnaeus, 1767) (Miridae) and *Nabis pseudoferus* Remane, 1949 (Nabidae), while Jerinić-Prodanović et al. (2010) reported seven species: *Anthocoris nemoralis* (Fabricius, 1794), *Anthocoris nemorum* (Linnaeus, 1761), *Orius (Heterorius) minutus* (Linnaeus, 1758) and *Orius (Orius) niger* Wolff, 1811 from the family Anthocoridae and *Campylomma verbasci* (Meyer-Dür, 1843), *Deraeocoris (Deraeocoris) ruber* (Linnaeus, 1758) and *Deraeocoris (Knightocapsus) lutescens* (Schilling, 1836) from the family Miridae.

There is no data on other predatory psyllid species in Serbia.

## Methods

Insect material was collected from 419 localities within the whole territory of the Republic of Serbia. Investigations were carried out in the period from 2005 to 2010, in field conditions and in the laboratory of the Faculty of Agriculture in Zemun, University of Belgrade. Locality mapping was carried out in World UTM (Universal Transverse Mercator) cartographic projection. Determination of coordinates of investigated localities in the field was carried out using GPS devices Geoexplorer 3 (Trimble) and E-trex Vista Hcx (Garmin), with an accuracy of 3 to 5 meters.

Adults of predatory true bugs were collected from psyllid colonies by an aspirator and their larvae were collected together with plant material and psyllids and further reared to adults in laboratory conditions in Petri dishes.

The species identification of Heteroptera was based on Wagner (1970–1971, 1975), Péricart (1972) and Kerzhner and Josifov (1999).

A part of the material is deposited in the first author’s collection in the Faculty of Agriculture, University of Belgrade, and another part, in the second author’s collection in Natural History Museum, Belgrade.

## Results and discussion

**Figure 1.**
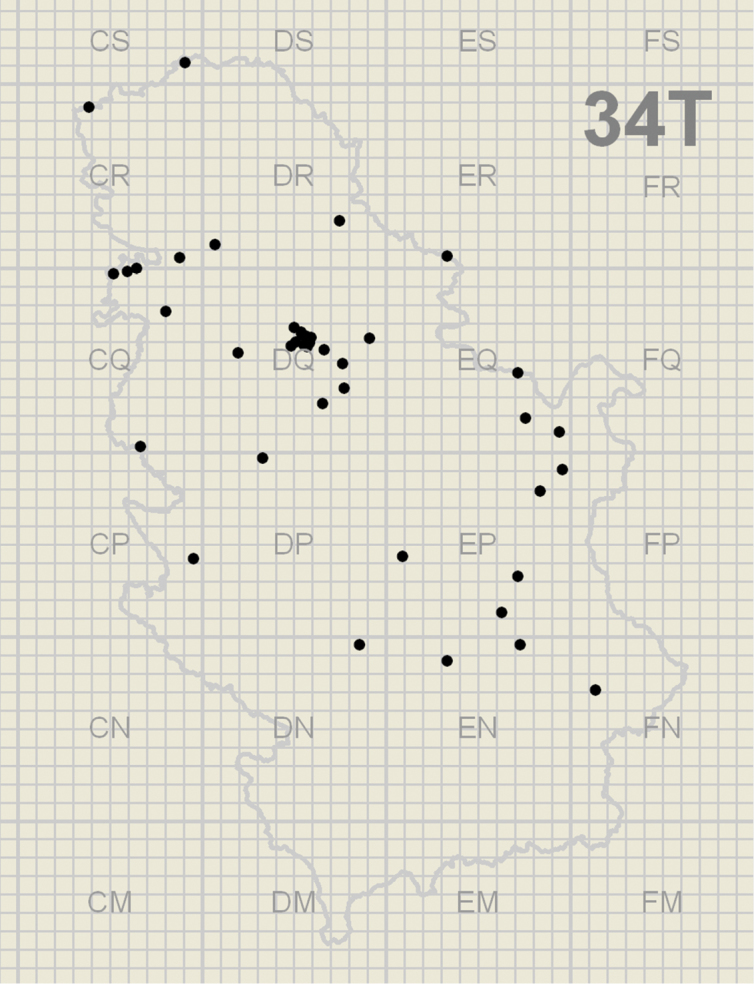
Localities in Serbia where true bug predators of psyllids were collected.

We collected and reared 21 true bug species predating on 28 psyllid species (Table 1) from 44 localities (Fig. 1 and Table 2). The identified true bugs belong to families Anthocoridae and Miridae.

**Table 1.** List of preys (Psylloidea) and their predators (Heteroptera) and host plants in Serbia.

**Preys (Psylloidea)**	**Predators (Heteroptera)**																				
	**Anthocoridae**							**Miridae**													
	**Ant amp**	**Ant con**	**Ant nea**	**Ant neu**	**Ori maj**	**Ori min**	**Ori nig**	**Atr mal**	**Cam ver**	**Der fla**	**Der lut**	**Der rub**	**Het gen**	**Hyp vis**	**Mal chl**	**Mir str**	**Ort mar**	**Psa ass**	**Psa fla**	**Psa que**	**Pse coc**
*Baeopelma foersteri*						Aln glu															
*Cacopsylla affinis*				Cra mon				Cra mon													
*Cacopsylla bidens*			Pyr com	Pyr com			Pyr com		Pyr com	Pyr com											
*Cacopsylla melanoneura*				Cra mon		Mal dom	Mal dom	Mal dom, Cra mon	Mal dom				Mal dom, Cra mon			Cra mon					
*Cacopsylla peregrina*				Cra mon				Cra mon								Cra mon					
*Cacopsylla picta*						Mal dom		Mal dom													
*Cacopsylla pulchra*				Sal pur																	
*Cacopsylla pyri*			Pyr com	Pyr com		Pyr com	Pyr com		Pyr com		Pyr com	Pyr com									
*Cacopsylla pyricola*									Pyr com												
*Cacopsylla pyrisuga*			Pyr com	Pyr com					Pyr com												
*Cacopsylla rhamnicola*						Rha cat					Rha cat						Rha cat				
*Cacopsylla visci*			Vis alb	Vis alb										Vis alb							
*Camarotoscena speciosa*			Pop nig			Pop nig					Pop nig										
*Craspedolepta* sp.							Art vul														
*Homotoma ficus*						Fic car				Fic car					Fic car						
*Livia junci*							Jun bul														
*Psylla buxi*	Bux sem	Bux sem		Bux sem																	
*Psyllopsis discrepans*			Fra spp		Fra spp	Fra orn				Fra ang								Fra spp	Fra spp	Fra orn, Fra spp	
*Psyllopsis fraxini*			Fra spp																		
*Psyllopsis fraxinicola*			Fra spp	Fra ang		Fra spp	Fra orn				Fra ang							Fra spp			Fra spp
*Psyllopsis machinosa*			Fra spp		Fra spp	Fra orn				Fra ang										Fra spp	
*Psyllopsis meliphila*			Fra spp																		
*Psyllopsis repens*			Fra spp			Fra orn															
*Trichochermes walkeri*							Rha cat														
*Trioza chenopodii*							Atr tat				Atr obl										
*Trioza mesembrina*						Cha hir															
*Trioza rhamni*						Rha cat				Rha cat											
*Trioza urticae*			Urt dio				Urt dio														

**Abbreviations<br/> Predators**. **Anthocoridae**: Ant amp, *Anthocoris amplicollis*; Ant con, *Anthocoris confusus*; Ant nea, *Anthocoris nemoralis*; Ant neu, *Anthocoris nemorum*; Ori maj, *Orius majusculus*; Ori min, *Orius minutus*; Ori nig, *Orius niger*; **Miridae**: Atr mal, *Atractotomus mali*; Cam ver, *Campylomma verbasci*; Der fla, *Deraeocoris flavilinea*; Der lut, *Deraeocoris lutescens*; Der rub, *Deraeocoris ruber*; Het gen, *Heterocordylus genistae*; Hyp vis, *Hypseloecus visci*; Mal chl, *Malacocoris chlorizans*; Mir str, *Miris striatus*; Ort mar, *Orthotylus marginalis*; Psa ass, *Psallus assimilis*; Psa fla, *Psallus flavellus*; Psa que, *Psallus quercus*; Pse coc, *Pseudoloxops coccinea*. <br/> **Host plants**: Aln glu, *Alnus glutinosa*; Art vul, *Artemisia vulgaris*; Atr obl, *Atriplex oblongifolia*; Atr tat, *Atriplex tatarica*; Bux sem, *Buxus sempervirens*; Cha hir, *Chaerophyllum hirsutum*; Cra mon, *Crataegus monogyna*; Fic car, *Ficus carica*; Fra ang, *Fraxinus angustifolia*; Fra orn, *Fraxinus ornus*; Fra spp, *Fraxinus* spp.; Jun bul, *Juncus bulbosus*; Mal dom, *Malus domestica*; Pop nig, *Populus nigra*; Pyr com, *Pyrus communis*; Rha cat, *Rhamnus cathartica*; Sal pur, *Salix purpurea*; Vis alb, *Viscum album*; Urt dio, *Urtica dioica*.

**Table 2.** Geographical coordinates of inspected localities.

**No**	**Locality**	**Latitude, Longitude**	**Altitude**
1	Bački breg	45°55'21"N, 18°55'24"E	90
2	Bavanište	44°48'42"N, 20°53'10"E	80
3	Beloljin	43°14'03"N, 21°24'26"E	290
4	Beograd–Autokomanda	44°47'20"N, 20°28'20"E	100
5	Beograd–Banjica	44°45'18"N, 20°28'58"E	190
6	Beograd–Block 45	44°47'36"N, 20°22'47"E	75
7	Beograd–Bulevar Aleksandra Karadjordjevića	44°46'50"N, 20°27'31"E	175
8	Beograd–Hotel Jugoslavija	44°49'36"N, 20°25'22"E	75
9	Beograd–Hram Svetog Save	44°47'53"N, 20°28'03"E	120
10	Beograd–Kalemegdan	44°49'19"N, 20°26'52"E	110
11	Beograd–Karaburma	44°48'48"N, 20°29'15"E	110
12	Beograd–Milošev konak	44°46'38"N, 20°25'36"E	80
13	Beograd–Voždovac	44°47'17"N, 20°28'28"E	85
14	Brestovačka Banja	44°03'36"N, 22°02'36"E	360
15	Dobra	44°38'23"N, 21°54'06"E	85
16	Draževac	43°28'08"N, 21°46'37"E	205
17	Galovica	44°46'22"N, 20°21'04"E	75
18	Grocka	44°41'21"N, 20°42'02"E	125
19	Ilinci	45°06'41"N, 19°07'16"E	80
20	Izvor	43°04'58"N, 22°23'57"E	290
21	Kelebija	46°08'59"N, 19°35'10"E	125
22	Kopaonik–Srebrenac	43°19'02"N, 20°50'08"E	1740
23	Klokočevac	44°20'53"N, 22°10'45"E	140
24	Koruška	45°11'46"N, 19°34'23"E	110
25	Lipovača	45°08'24"N, 19°16'53"E	165
26	Luka	44°09'46"N, 22°11'56"E	340
27	Majdanpek	44°25'10"N, 21°57'10"E	520
28	Nemenikuće	44°29'38"N, 20°34'00"E	280
29	Niš	43°18'35"N, 21°53'50"E	200
30	Novi Sad–Detelinara	45°15'50"N, 19°48'56"E	80
31	Obedska bara	44°44'10"N, 19°59'15"E	80
32	Oparić	43°44'40"N, 21°06'38"E	310
33	Radenković	44°56'01"N, 19°29'05"E	80
34	Radmilovac	44°45'15"N, 20°34'39"E	160
35	Sokobanja	43°38'41"N, 21°53'11"E	350
36	Sutjeska	45°23'02"N, 20°41'53"E	75
37	Šid	45°07'31"N, 19°12'58"E	105
38	Umčari	44°34'10"N, 20°43'00"E	160
39	Uzovnica	44°16'12"N, 19°19'47"E	170
40	Veliko Središte	45°12'54"N, 21°25'30"E	120
41	Vrujci	44°13'26"N, 20°09'53"E	170
42	Zemun–Nova Galenika	44°51'41"N, 20°22'11"E	90
43	Zemunski kej	44°50'29"N, 20°25'06"E	75
44	Zlatibor–Kraljevske Vode	43°43'39"N, 19°42'06"E	950

### Anthocoridae

#### 1) Anthocoris amplicollis

(Horváth, 1839)

##### Trophic status.

Zoophagous.

##### Distribution.

Europe.

##### Prey.

***Psylla buxi* (Linnaeus, 1758)**, from *Buxus sempervirens*, Nova Galenika, 13.VI.2009, reared 3♂♂, 2♀♀.

*Anthocoris amplicollis* was already reported in Serbia (Protić and Stojanović 2003) but the above mentioned record is the first one in Serbia as a psyllid predator. In Switzerland, it was registered by Wyniger and Burckhardt (2003) in galls of *Psyllopsis fraxini*. According to available literature data, *Anthocoris amplicollis* has not been published as a predator of *Psylla buxi*.

#### 2) Anthocoris confusus

Reuter, 1884

##### Trophic status.

Zoophagous.

##### Distribution.

Palaearctic.

##### Prey.

***Psylla buxi***, from *Buxus semprevirens*, Sokobanja, 25.IX.2009, reared 1♀.

Registered as a psyllid predator on conifers (Wyniger and Burckhardt 2003) and aphids (Herard 1986). In the present paper, reported for the first time as a psyllid predator in Serbia.

#### 3) Anthocoris nemoralis

(Fabricius, 1794)

##### Trophic status.

Zoophagous.

##### Distribution.

Euro-Mediterranean.

##### Preys.

***Cacopsylla bidens* (Šulc, 1907)**, from *Pyrus communis*, Beograd–Karaburma, 19.V.2006, reared 2♀♀ ‘ex larva' 25.V.2006. ***Cacopsylla pyri***, from *Pyrus communis*, Nemenikuće, 15.VI.2006, reared 1♂ ‘ex larva' 22.VI.2006; Radmilovac, 2.IX.2005, reared 1♂; 12.VI.2006, reared 1♂ ‘ex larva' 25.VI.2006; 26.X.2006, collected 1♂; 7.VI.2007, reared 1♂, 1♀ ‘ex larva' 11.VI.2007, 20.VI.2007, collected 1 larva and 1♂; 26.X.2008, reared 1♂ ‘ex larva' 3.XI.2008. ***Cacopsylla pyrisuga***, from *Pyrus communis*, Zemunski kej, 28.V.2007, collected 1♀. ***Cacopsylla visci* (Curtis, 1835)**, from *Viscum album*, Beograd–Bulevar Aleksandra Karađorđevića, 25.III.2007, reared 2♂♂, 1♀ ‘ex larva' 12.IV.2007; 23.IV.2007, reared 2♂♂, 5♀♀ ‘ex larva' 3.V.2007. ***Camarotoscena speciosa* (Flor, 1861)**, from *Populus nigra*, Radmilovac, 22.V.2000, reared 1♂ ‘ex larva' 25.V.2000; Zemun–Nova Galenika, 25.VIII.2008, collected 1♀; 1.IX.2008, reared 3♀♀ ‘ex larva' 9.IX.2008; 10.IX.2008, collected 1♀, 1♂; 17.IX.2008, collected 1♀, 1♂; 24.IX.2008, reared 2♂♂, 2♀♀ ‘ex larva' 1.X.2008; 27.VII.2010, collected 1♂, 1♀, 18.X.2010, collected 1♂, 1♀. ***Psyllopsis discrepans* (Flor, 1861)**, from *Fraxinus* sp., Beograd–Autokomanda, 10.V.2007, reared 1♀; Brestovačka Banja, 25.V.2007, reared 5♂♂, 4♀♀ ’ex larva'8.VI.2007; Ilinci, 24.V.2008, collected 1♂; Majdanpek, 25.V.2007, reared 1♂, 1♀ ‘ex larva' 3.VI.2007, Milošev konak, 21.VI.2007, 1♂. ***Psyllopsis fraxini***, from *Fraxinus* sp., Veliko Središte, 30.V.2006, reared 2♀♀. ***Psyllopsis fraxinicola* (Foerster, 1848)**, from *Fraxinus* sp., Beograd–Autokomanda, 10.V.2007, reared 1♀; Beograd–Kalemegdan, 24.V.2007, collected 1♂; Brestovačka Banja, 25.V.2007, reared 5♂♂, 4♀♀ ‘ex larva' 8.VI.2007; Majdanpek, 25.V.2007, reared 1♂, 1♀ ‘ex larva' 3.VI.2007; Veliko Središte, 4.VI.2006, reared 2♀♀ ‘ex larva' 19.VI.2006. ***Psyllopsis machinosa* Loginova, 1963**, from *Fraxinus* sp., Beograd–Autokomanda, 10.V. 2007, reared 1♀. ***Psyllopsis meliphila* Löw, 1881**, from *Fraxinus* sp., Nemenikuće, 15.VI.2006, collected 1♂. ***Psyllopsis repens* Loginova, 1963**, from *Fraxinus* sp., Beograd–Autokomanda, 14.X.2008, reared 1♀. ***Trioza urticae* (Linnaeus, 1758)**, from *Urtica dioica*, Ilinci, 24.VI.2007, collected 2♀♀.

*Anthocoris nemoralis* is an important component of the natural enemy community in pear and apple orchards where it provides biological control against arthropod pests, particularly psyllids (Horton et al. 2004). Investigating the predator–prey complex of *Cacopsylla pyrisuga* in a pear orchard in France, Herard (1986) found that *Anthocoris nemoralis* was the most efficient enemy against this pest. *Anthocoris nemoralis* is mentioned in many papers as a permanent member of biocomplexes of pear psyllids in Europe (Wheeler 2000b, Erler 2004, Sigsgaard et al. 2006). In Turkey, *Anthocoris nemoralis* was an equally present and efficient predator of pear psyllid *Cacopsylla pyrisuga*, both in treated and untreated orchards, but still insufficient for its full control (Erler 2004). In Spain, Jauset et al. (2006) determined *Anthocoris nemoralis* as a very efficient predator of *Cacopsylla pyrisuga*, both in treated and untreated pear orchards. Now, there is a mass production of *Anthocoris nemoralis* in companies specialized for biological control of harmful insects (Sigsgaard et al. 2006). The same authors reported that *Anthocoris nemoralis* mostly prefers *Cacopsylla pyrisuga* to aphids, and that it prefers laying eggs on pear to apple. *Anthocoris nemoralis* is a polyphagous predatory species having psyllids as a usual prey.

It is distributed in Europe and the Mediterranean. From Europe it was introduced into North America in 1963 in order to control pear psyllid *Cacopsylla pyricola*, giving satisfactory results (Horton et al. 2004). This species has adapted to this region so well that it has suppressed autochthonous predatory species *Anthocoris antevolens* and *Anthocoris melanocerus* (Herard 1986, Horton et al. 2004). In Serbia, in a pear orchard, Pavićević (1977), Grbić et al. (1989) and Jerinić-Prodanović (2010) note a permanent presence of *Anthocoris nemoralis*, both during vegetation and winter period together with an overwintering adult of *Cacopsylla pyrisuga*. *Anthocoris nemoralis* was also reported as a predator of *Psyllopsis repens* in Serbia (Malenovský and Jerinić-Prodanović 2011).

#### 4) Anthocoris nemorum

(Linnaeus, 1761)

##### Trophic status.

Zoophagous.

##### Distribution.

Eurosiberian.

##### Preys.

***Cacopsylla affinis* (Löw, 1880)**, from *Crataegus monogyna*, Ilinci, 27.IV.2008, reared 1♂ ‘ex larva' 14.V. 2008. ***Cacopsylla bidens***, from *Pyrus communis*, Nemenikuće, 15.VI.2006, reared 1♀. ***Cacopsylla melanoneura* (Foerster, 1848)**, from *Crataegus monogyna*, Ilinci, 27.IV.2008, reared 1♂ ‘ex larva' 14.V.2008; Klokočevac, 10.V.2008, collected 1♀. ***Cacopsylla peregrina* (Foerster, 1848)**, from *Crataegus monogyna*, Ilinci, 27.IV.2008, reared 1♂ ‘ex larva' 14.V.2008. ***Cacopsylla pulchra* (Zetterstedt, 1838)**, from *Salix purpurea*, Zlatibor–Kraljevske Vode, 30.IV.2007, collected 1♀ and 1 larva. ***Cacopsylla pyri***, from *Pyrus communis*, Novi Sad–Detelinara, 23.V.2008, reared 2♂♂, 3♀♀ ‘ex larva' 4.VI.2008; 14.VI.2008, reared 3♂♂ ‘ex larva' 17.VI.2008. ***Cacopsylla pyrisuga***, from *Pyrus communis*, Grocka, 10.V.2008, reared 1♀ ‘ex larva' 23.V.2008. ***Cacopsylla visci***, from *Viscum album*, Beograd–Bulevar Aleksandra Karađorđevića, 23.IV.2007, reared 1♂ ‘ex larva' 3.V.2007. ***Psylla buxi***, from *Buxus sempervirens*, Vrujci, 1.VI.2009, collected 2♂♂, 1♀; Šid, 3.V.2008, collected 2♀♀; Zemun–Nova Galenika, 4.V.2008, reared 2♂♂, 3♀♀ ‘ex larva' 15.V.2008; 30.VII.2008, collected 1♀; 14.VI.2009, collected 2 specimens. ***Psyllopsis fraxinicola***, from *Fraxinus angustifolia*, Beograd–Hram Svetog Save, 14.IV.2008, reared 1♂ and 1 larva.

*Anthocoris nemorum* is noted as a predator of many insect species, in the first place Hemiptera, Diptera, eggs of Lepidoptera and mites (Herard 1986, Wheeler 2000b, Sigsgaard et al. 2006), already registered as a predator of both *Cacopsylla pyrisuga* and *Psyllopsis fraxini* (Herard 1986). It is also largely reported as an efficient predator of apple psyllid *Cacopsylla mali* in Norway (Jonsson 1983). In England, Hodkinson and Flint (1971) determined *Anthocoris nemorum* as a predator of *Psyllopsis fraxini* collected from ash, while in Germany Novak and Achtziger (1995) registered it as a predator of hawthorn psyllids *Cacopsylla melanoneura* and *Cacopsylla peregrina*. Sigsgaard et al. (2006) note *Anthocoris nemorum* as a more polyphagous species than *Anthocoris nemoralis*. They also determined in experimental conditions that *Anthocoris nemorum* prefers aphids to psyllids, and has a preference for laying eggs on apple rather than on pear.

*Anthocoris nemorum* is an Eurosiberian species, introduced to North America in order to control *Cacopsylla pyricola* just like *Anthocoris nemoralis*, but without satisfactory results (Herard 1986).

*Anthocoris nemorum* is reported here for the first time as a predator of psyllids in Serbia.

#### 5) Orius (Heterorius) majusculus

Reuter, 1884

##### Trophic status.

Zoophagous.

##### Distribution.

Euro-Atlantic.

##### Preys.

***Psyllopsis discrepans*** and ***Psyllopsis machinosa***, from *Fraxinus* spp., Beograd–Autokomanda, 6.V.2009, collected 1♂.

*Orius majusculus* was registered as a predator of psyllids (Herard 1986). It is noted as a predator of aphids, such as *Diuraphis noxia* and *Schizaphis graminum* in Russia, mites in France, whiteflies in greenhouses in Italy and pear psyllid *Cacopsylla pyri* in France (Péricart 1972, Herard 1986).

The present paper reports *Orius majusculus* as a psyllid predator for the first time in Serbia and *Psyllopsis discrepans* and *Psyllopsis machinosa* for the first time as a prey of *Orius majusculus*.

#### 6) Orius (Heterorius) minutus

(Linnaeus, 1758)

Fig. 2

##### Trophic status.

Zoophagous.

##### Distribution.

Palaearctic.

##### Preys.

***Baeopelma foersteri* (Flor, 1861)**, from *Alnus glutinosa*, Radenković, 3.VI.2006, collected 1♀. ***Cacopsylla melanoneura***, from *Malus domestica*, Beograd–Hotel Jugoslavija, 26.V.2005, reared 1♂; Ilinci, 2.V.2010, reared 1♂, 2♀♀ ‘ex larva' 10.V.2010. ***Cacopsylla picta* (Foerster, 1848)**, from *Malus domestica*, Beograd–Hotel Jugoslavija, 26.V.2005, reared 1♂. ***Cacopsylla pyri***, from *Pyrus communis*, Radmilovac, 10.VII.2006, reared 1♂, 2♀♀ ‘ex larva' 20.VII.2006; 26.VII.2006, collected 1♂; 4.IX.2006, collected 1♂. ***Cacopsylla rhamnicola* (Scott, 1876)**, from *Rhamnus cathartica*, Kelebija, 25.V.2005, reared 1♀ ‘ex larva' 6.VI.2005. ***Camarotoscena speciosa***, from *Populus nigra*, Zemun–Nova Galenika, 1.IX.2008, reared 1♂ ‘ex larva' 9.IX.2008; 24.IX.2008, reared 1♂ ‘ex larva' 1.X.2008. ***Homotoma ficus* (Linnaeus, 1758)**, from *Ficus carica*, Beograd–Banjica, 23.IX.2008, collected 2♂♂, 3♀♀, feeding on eggs. ***Psyllopsis discrepans***, from *Fraxinus ornus*, Ilinci, 21.V.2005, collected 1♂. ***Psyllopsis discrepans*** and ***Psyllopsis repens***, from *Fraxinus ornus*, Beograd–Autokomanda, 7.IX.2008, reared 5♂♂, 4♀♀ ‘ex larva' 11.IX.2008; 21.IX.2008, collected 3♂♂; 23.IX.2008, collected 1♂; 7.X.2008, collected 1♂, 1♀; 14.X.2008, collected 1♀; 21.X.2008, collected 1♀. ***Psyllopsis fraxinicola***, from *Fraxinus* sp., Vrujci, 1.VII.2009, 1♀. ***Psyllopsis machinosa***, from *Fraxinus ornus*, Beograd–Autokomanda, 6.V.2009, reared 1♀ ‘ex larva' 10.V.2009. ***Psyllopsis repens***, from *Fraxinus ornus*, Beograd–Autokomanda, 21.VIII.2010, reared 1♀. ***Trioza mesembrina* Burckhardt, 1986**, from *Chaerophyllum hirsutum*, Kopaonik–Srebrenac, 7.VIII.2008, reared 1♂. ***Trioza rhamni* (Schrank, 1801)**, from *Rhamnus cathartica*, Kelebija, 25.V.2005, reared 1♀ ‘ex larva' 6.VI.2005; Ilinci, 2.V.2009, 1♂.

*Orius minutus* is an extremely polyphagous species distributed in Europe, Siberia, China and Mediterranean region. Many authors determined it as a predator of harmful insect species from a number of orders (Thysanoptera, Diptera, Lepidoptera, Coleoptera and Hemiptera Homoptera). Already reported as a psyllid predator (Herard 1986). In France, Herard (1986) determined *Orius minutus* as a predator of pear psyllids, primarily *Cacopsylla pyrisuga*, and hawthorn psyllids. Also in Slovenia, Vrabl and Matis (1977) register it as a predator of *Cacopsylla pyrisuga* and *Cacopsylla pyrisuga*. In Serbia, Pavićević (1977) and Jerinić-Prodanović et al. (2010) determined *Orius minutus* as a predator of *Cacopsylla pyrisuga* in pear orchards. Malenovský and Jerinić-Prodanović (2011) also found it as a predator of *Psyllopsis repens*. In Croatia, Arčanin and Balarin (1972) recognized the significance of *Orius minutus* in the reduction of the mite *Panonychus ulmi*.

**Figure 2.**
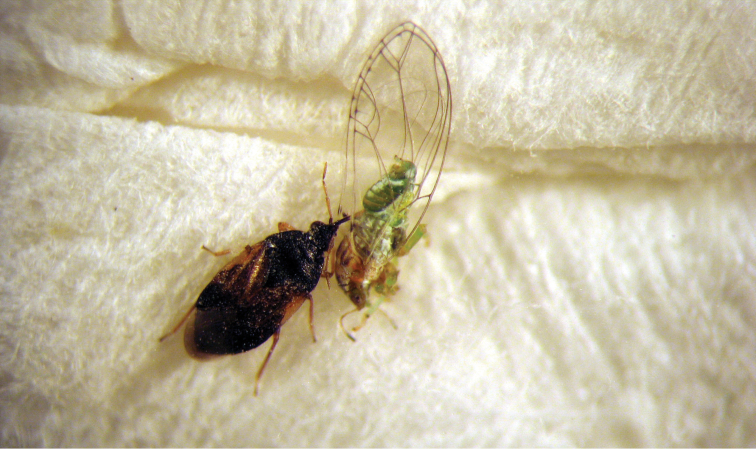
*Orius minutus* feeding on *Trioza rhamni*.

#### 7) Orius (Orius) niger

Wolff, 1811

##### Trophic status.

Zoophagous.

##### Distribution.

Palaearctic.

##### Preys.

***Cacopsylla bidens***, from *Pyrus communis*, Ilinci, 13.X.2008, collected 1♂. ***Cacopsylla melanoneura***, from *Malus domestica*, Lipovača, 29.IV.2006, collected 1♂. ***Cacopsylla pyri***, from *Pyrus communis*, Radmilovac, 26.VI.2006, reared 1♂ ‘ex larva' 30.VI.2006; 10.VII.2006, reared 1♀ ‘ex larva' 20.VII.2006. ***Craspedolepta* sp.**, from *Artemisia vulgaris*, Sutjeska, 2.X.2009, reared 1♂ and 4♀♀. ***Psyllopsis fraxinicola***, from *Fraxinus ornus*, Vrujci, 30.VI.2009, collected 1 specimen. ***Livia junci* (Schrank, 1789)**, from *Juncus bulbosus*, Beograd–Block 45, 10.VIII.2005, reared 1♂ ‘ex larva' 16.VIII.2005. ***Trichochermes walkeri* (Foerster, 1848)**, from *Rhamnus cathartica*, Ilinci, 14.IX.2008, 2♂♂, 2♀♀. ***Trioza chenopodii* Reuter, 1876**, from *Atriplex tatarica*, Ilinci, 20.VIII.2006, 1♂ ‘ex larva' 23.VIII.2006. ***Trioza urticae***, from *Urtica dioica*, Bački breg, 7.VI.2005, reared 1♂; Ilinci, 14.V.2005, reared 1♂, 1♀ ‘ex larva' 27.V.2005; Luka, 25.V.2007, reared 1♂.

*Orius niger* is widespread in Western Palaearctic, very rare in the Mediterranean region, also reported from China. It is a very polyphagous species, preying on aphids, psyllids, whiteflies, thrips, larvae of noctuids, mites (Péricart 1972, Herard 1986, Protić 1993).

In south France, Herard (1986) determined *Orius niger* on pears as a predator of *Cacopsylla pyri*, but also collected it in a large number from *Trioza urticae* from nettle, which was surrounding the pear orchards. In Croatia, *Orius niger* was determined along with *Orius minutus* on *Panonychus ulmi* in an apple orchard (Arčanin and Balarin 1972).

In Serbia, Grbić et al. (1989), investigating pear psyllid predators, reported *Orius* spp., so we are not able to compare our results with theirs. In the same paper, authors mentioned the presence of other *Orius* species frequently during summer and autumn which is in accordance with our investigations.

### Miridae

#### 8) Atractotomus mali

Meyer-Dür, 1843

##### Trophic status.

Phytozoophagous.

##### Distribution.

Palaearctic.

##### Preys.

***Cacopsylla affinis***, from *Crataegus monogyna*, Koruška, 1.V.2008, reared 1♀ ‘ex larva' 8.V.2008. ***Cacopsylla melanoneura***, from *Crataegus monogyna*, Beograd–Hotel Jugoslavija, 22.IV.2008, reared 2♂♂, 1♀ ‘ex larva' 30.IV.2008; 18.V.2008, reared 1♀ ‘ex larva' 23.V.2008; Dobra, 10.V.2008, reared 1♂, 1♀ ‘ex larva' 21.V.2008; Draževac, 20.IV.2008, reared 1♂, 1♀ 2.V.2008; Koruška, 1.V.2008, reared 1♀ ‘ex larva' 8.V.2008. ***Cacopsylla melanoneura***, from *Malus domestica*, Ilinci, 24.V.2008, reared 1♀ ‘ex larva' 30.V.2008; Oparić, 4.V.2008, reared 2♂ ‘ex larva' 19.V.2008; Ilinci, 2.V.2010, reared 1♀. ***Cacopsylla peregrina***, from *Crataegus monogyna*, Beograd–Hotel Jugoslavija, 22.IV.2008, reared 2♂♂, 1♀ ‘ex larva' 30.IV.2008; Dobra, 10.V.2008, reared 1♂, 1♀ ‘ex larva' 21.V.2008; Koruška, 1.V.2008, reared 1♀ ‘ex larva' 8.V.2008. ***Cacopsylla picta***, from *Malus domestica*, Ilinci, 24.V.2008, 1♀ ‘ex larva' 30.V.2008.

*Atractotomus mali* has been reported so far as a predator of mites, aphids, thrips, psyllids, butterfly larvae and pupae (Wheeler 2000b). It was registered as a predator of apple psyllid *Cacopsylla mali* in Norway (Jonsson 1983) and of pear psyllid *Cacopsylla pyrisuga* in Greece (Santas 1987). In Germany, Novak and Achtziger (1995) registered it as a predator of hawthorn psyllids *Cacopsylla* spp.

First record of *Atractotomus mali* as a predator of *Cacopsylla picta*. The above mentioned data are the first ones for *Atractotomus mali* as a psyllid predator in Serbia.

#### 9) Campylomma verbasci

(Meyer-Dür, 1843)

##### Trophic status.

Zoophytophagous.

##### Distribution.

Holarctic.

##### Preys.

***Cacopsylla bidens***, from *Pyrus communis*, Ilinci, 24.V.2008, reared 1♂ ‘ex larva' 29.V.2008; Bavanište, 25.V.2006, reared 1♂, 1♀ ‘ex larva' 30.V.2006. ***Cacopsylla pyri***, ***Cacopsylla pyricola*** and ***Cacopsylla pyrisuga***, from *Pyrus communis*, Bavanište, 25.V.2006, 1♂, 1♀ ‘ex larva' 30.V. 2006. ***Cacopsylla melanoneura***, from *Malus domestica*, Ilinci, 2.V.2010, reared 2♂♂.

*Campylomma verbasci* is a zoophytophagous species preying on apple aphids, pear psyllids, codling moth, thrips and mites (Wheeler 2000b). Its most common prey among insects are *Aphis pomi* and *Cacopsylla mali*, and among mites *Panonychus ulmi* and *Tetranychus urticae* (Hagen et al. 1999, Wheeler 2000b, Bradley 2007).

However, if there is a lack of prey, it can feed on apple fruits, rarely pear, causing the harm to their aesthetic value. Therefore, *Campylomma verbasci* is a significant fruit pest in Canada (Hagen et al. 1999, Wheeler 2000a, Bradley 2007). Erler (2004) reported the presence of *Campylomma verbasci* as a predator of *Cacopsylla pyrisuga* in treated and untreated pear orchards in Turkey, and Harizanova et al. (2012) mentioned it on *Acizzia jamatonica* in Bulgaria.

Already known in Serbia (Protić 1993) but in our investigations registered for the first time as a predator of psyllids in this country.

#### 10) Deraeocoris (Deraeocoris) flavilinea

(A. Costa, 1862)

##### Trophic status.

Zoophytophagous.

##### Distribution.

Western and Central Europe.

##### Preys.

***Cacopsylla bidens***, from *Pyrus communis*, Beograd–Karaburma, 4.V.2006, reared 1♀ ‘ex larva' 18.V.2006. ***Homotoma ficus***, from *Ficus carica*, Beograd–Banjica, 21.V.2009, collected 1♂, 2♀♀. ***Trioza rhamni***, from *Rhamnus cathartica*, Beograd–Hotel Jugoslavija, 26.V.2005, reared 1♂ ‘ex larva' 29.V.2005. ***Psyllopsis discrepans*** and ***Psyllopsis machinosa***, from *Fraxinus angustifolia*, Beograd–Autokomanda, 21.V.2009, 1♂, 1♀.

*Deraeocoris flavilinea* is reported so far as a predator of psyllids (Jerinić-Prodanović and Protić 2011, Simov et al. 2012). Until 1980’s, it was known only from Sicily, from where it has spread to Central Europe where it is now considered as an invasive species (Rabitsch 2008). As a predator of psyllids, it has been known in Serbia since 2011 (Jerinić-Prodanović and Protić 2011).

#### 11) Deraeocoris (Deraeocoris) ruber

(Linnaeus, 1758)

##### Trophic status.

Zoophytophagous.

##### Distribution.

Holarctic.

##### Prey.

***Cacopsylla pyri***, from *Pyrus communis*, Radmilovac, 10.VII.2006, reared 1♀.

A very polyphagous zoophytophagous species. A Holarctic species occurring in large quantities in the south of Europe.

Already mentioned as a predator of *Cacopsylla pyrisuga* (Herard 1986). It also preys on younger caterpillar instars of some butterflies, mites and various other small insects in apple orchards, on *Rubus* spp. and *Urtica* spp. as well as on aphids on *Corylus* spp. (Herard 1986).

Reported as a predator of *Acizzia jamatonica* (Harizanova et al. 2012) in Bulgaria and *Cacopsylla pyrisuga* in Serbia (Jerinić-Prodanović et al. 2010).

#### 12) Deraeocoris (Knightocapsus) lutescens

(Schilling, 1836)

##### Trophic status.

Zoophagous.

##### Distribution.

Euro-Mediterranean.

##### Preys.

***Cacopsylla pyri***, from *Pyrus communis*, Izvor, 14.IV.2009, 1♂. ***Cacopsylla rhamnicola***, from *Rhamnus cathartica*, Obedska bara, 4.VI.2005, reared 1♂ ‘ex larva' 18.VI.2005. ***Camarotoscena speciosa***, from *Populus nigra*, Zemun–Nova Galenika, 18.X.2010, collected 1♀. ***Psyllopsis fraxinicola***, from *Fraxinus angustifolia*, Zemun–Nova Galenika, 1.IX.2006, collected 1♀. ***Trioza chenopodii***, from *Atriplex oblongifolia*, Galovica, 18.VII.2003, reared 1♂.

*Deraeocoris lutescens* is a Mediterranean species, distributed also in small numbers in Central Europe. Known mainly as an egg predator of pear psyllid *Cacopsylla pyrisuga* and hawthorn psyllid *Cacopsylla crataegi* (Herard 1986). It is also reported as a predator of aphids and mite *Panonychus ulmi* in apple orchards in Croatia (Arčanin and Balarin 1972) and in pear orchards as a predator of *Cacopsylla pyrisuga* in France and Turkey (Herard 1986, Erler 2004).

*Deraeocoris lutescens* has been already registered in Serbia (Protić 1993) but here is reported for the first time as a predator of psyllids in this country.

#### 13) Heterocordylus (Heterodactylus) genistae

(Scopoli, 1763)

##### Trophic status.

Phytozoophagous.

##### Distribution.

Europe.

##### Prey.

***Cacopsylla melanoneura***, from *Malus domestica*, Beloljin, 4.V.2008, collected 1♂; Ilinci, 20.V.2006, reared 1♂ ‘ex larva' 25.V.2006; Uzovnica, 29.IV.2007, collected 1 specimen.

*Heterocordylus genistae* is mentioned in the literature as a beneficial insect being a predator of psyllids both in larval and adult stage. It is registered as a predator of various other insects (Protić 1993, 1998).

In the present paper, we report *Heterocordylus genistae* for the first time as a predator of psyllids in Serbia.

#### 14) Hypseloecus visci

(Puton, 1888)

##### Trophic status.

Zoophagous.

##### Distribution.

Europe.

##### Prey.

***Cacopsylla visci***, from *Viscum album*, Beograd–Bulevar Aleksandra Karađorđevića, 25.III.2007, reared 4♀♀ ‘ex larva' 16.IV.2007.

An exclusively zoophagous species.

Already known from Serbia as a psyllid predator (Jerinić-Prodanović and Protić 2011).

#### 15) Malacocoris chlorizans

Panzer, 1794

##### Trophic status.

Zoophagous.

##### Distribution.

Eurasia.

##### Prey.

***Homotoma ficus***, from *Ficus carica*, Beograd–Hotel Jugoslavija, 16.V.2007, collected 1♂, 1♀; Beograd–Voždovac, 26.V.2007, collected 5♂♂, 1♀; Zemunski kej, 15.V.2008, collected 1 larva.

A general predator on aphids, psyllids, eggs and larvae of leaf miner moths (Wheeler 2000b, Wyniger and Burckhardt 2003). In Croatia, it is registered as a predator of *Panonychus ulmi* in apple orchards by Arčanin and Balarin (1972).

*Malacocoris chlorizans* has been already registered in Serbia (Protić 1998) but in the present paper is reported for the first time as a predator of psyllids in this country.

#### 16) Miris striatus

(Linnaeus, 1758)

##### Trophic status.

Zoophagous.

##### Distribution.

Europe, Central Asia.

##### Preys.

***Cacopsylla melanoneura*** and ***Cacopsylla peregrina***, from *Crataegus monogyna*, Dobra, 10.V.2008, reared 1♀ ‘ex larva' 21.V.2008.

Already reported from Serbia (Protić 1993, 1998). The above mentioned record is the first one of *Miris striatus* as a predator of psyllids.

#### 17) Orthotylus (Orthotylus) marginalis

Reuter, 1884

##### Trophic status.

Zoophytophagous.

##### Distribution.

Eurosiberian.

##### Prey.

***Cacopsylla rhamnicola***, from *Rhamnus cathartica*, Beograd–Hotel Jugoslavija, 15.V.2008, reared 1♀ ‘ex larva' 19.V.2008.

*Orthotylus marginalis* is registered as a predator of aphids and psyllids (Wheeler 2000b). In Finland and Russia, it was mentioned as a predator of *Cacopsylla mali* (Jonsson 1983).

Registered in Serbia (Protić 2011) but here reported for the first time as a psyllid predator.

#### 18) Psallus (Hylopsallus) assimilis

Stichel, 1956

##### Trophic status.

Phytozoophagous.

##### Distribution.

Europe.

##### Preys.

***Psyllopsis discrepans***, from *Fraxinus* sp., Ilinci, 21.V.2005, reared 2♂♂, 2♀♀; Umčari, 25.V.2007, reared 1♀. ***Psyllopsis fraxinicola*** and ***Psyllopsis discrepans***, from *Fraxinus* sp., Beograd–Autokomanda, 10.V.2007, reared 5♂♂, 8♀♀ ‘ex larva' 16.V.2007.

Already known as a predator of various insect species, inlcuding psyllids.

Previously registered in Serbia (Protić 1998) but in the present paper reported for the first time as a psyllid predator in this country.

#### 19) Psallus (Psallus) flavellus

Stichel, 1933

Fig. 3

##### Trophic status.

Phytozoophagous.

##### Distribution.

Europe.

##### Preys.

***Psyllopsis* spp.**, from *Fraxinus* sp., Beograd–Autokomanda, 8.V.2010, reared 3♂♂, 1♀. ***Psyllopsis discrepans***, from *Fraxinus* sp., Beograd–Autokomanda, 13.V.2010, reared 2♂♂, 1♀.

Previously registered in Serbia (Protić 2011). Reported here for the first time as a psyllid predator.

**Figure 3.**
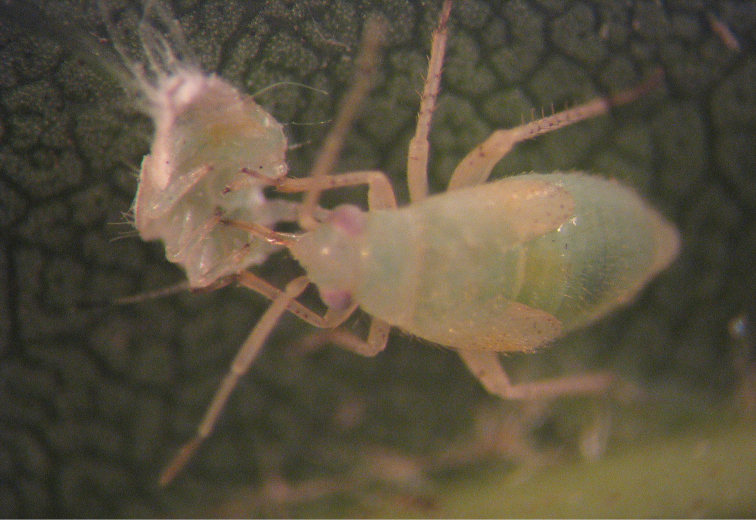
Larva of *Psallus flavellus* feeding on *Psyllopsis fraxinicola*.

#### 20) Psallus (Phylidea) quercus

Kirschbaum, 1856

##### Trophic status.

Phytozoophagous.

##### Distribution.

Europe, Asia.

##### Preys.

***Psyllopsis discrepans***, from *Fraxinus ornus*, Ilinci, 24.V. 2008, collected 1♀; 17.V. 2009, 1♂, 2♀♀. ***Psyllopsis discrepans*** and ***Psyllopsis machinosa***, from *Fraxinus* sp.,Beograd–Autokomanda, 6.V.2009, reared 4♀♀ ‘ex larva' 10.V.2009; 21.V.2009, reared 1♂, 3♀♀.

So far known as a predator of aphids, psyllids, thrips, spiders and eggs of various insects (Protić 1998).

Registered in Serbia (Protić 2011) but here reported for the first time as a psyllid predator in this country.

#### 21) Pseudoloxops coccinea

Meyer-Dür, 1843

##### Trophic status.

Zoophagous.

##### Distribution.

Euro-Mediterranean.

##### Preys.

***Psyllopsis fraxinicola***, from *Fraxinus* sp., Niš, 27.V.2008, collected 1♂. ***Psyllopsis* sp.**, *Fraxinus* sp., Beograd–Autokomanda 8.V.2010, reared 1♂ ‘ex larva' 21.V.2010.

Registered in Serbia (Protić 1999). Reported for the first time as a predator of psyllids.

## Conclusions

From 28 psyllid species and 19 host plants, we reared or collected 21 species of true bugs belonging to the families Anthocoridae and Miridae. According to available literature data, 12 of the recorded species are zoophagous, while the other nine have mixed nutrition.

*Miris striatus*, *Pseudoloxops coccinea* and *Psallus flavellus* (Miridae) have not been registered as psyllid predators so far. Sixteen species of true bugs are recorded here for the first time as psyllid predators in Serbia (*Anthocoris amplicollis*, *Anthocoris nemorum*, *Anthocoris confusus*, *Orius majusculus*, *Orius niger*, *Atractotomus mali*, *Campylomma verbasci*, *Deraeocoris lutescens*, *Heterocordylus genistae*, *Malacocoris chlorizans*, *Orthotylus marginalis*, *Psallus assimilis*, *Psallus quercus*, *Psallus flavellus*, *Miris striatus* and *Pseudoloxops coccinea*).

From the family Anthocoridae, we identified seven species: *Anthocoris amplicollis*, *Anthocoris confusus*, *Anthocoris nemoralis*, *Anthocoris nemorum*, *Orius majusculus*, *Orius minutus* and *Orius niger*. The most polyphagous among them was *Orius minutus*, found on 13 species of psyllids: *Baeopelma foersteri*, *Cacopsylla melanoneura*, *Cacopsylla picta*, *Cacopsylla pyrisuga*, *Cacopsylla rhamnicola*, *Camarotoscena speciosa*, *Homotoma ficus*, *Psyllopsis discrepans*, *Psyllopsis fraxinicola*, *Psyllopsis machinosa*, *Psyllopsis repens*, *Trioza mesembrina* and *Trioza rhamni*.

From the family Miridae, we reared or collected 14 species: *Atractotomus mali*, *Campylomma verbasci*, *Deraeocoris flavilinea*, *Deraeocoris ruber*, *Deraeocoris lutescens*, *Heterocordylus genistae*, *Hypseloecus visci*, *Malacocoris chlorizans*, *Miris striatus*, *Orthotylus marginalis*, *Psallus assimilis*, *Psallus flavellus*, *Psallus quercus* and *Pseudoloxops coccinea*. Among them, the most polyphagous were *Campylomma verbasci*, *Deraeocoris flavillinea* and *Deraeocoris lutescens*, each registered on five psyllid species.

Most of predatory true bugs are registered on deciduous perennial plants. We found the highest number of predatory true bugs on psyllids which overwinter on host plant and have more than one generation per year, e.g. *Cacopsylla pyri*, *Psyllopsis fraxinicola* and *Psyllopsis discrepans*. On each of them, seven predatory true bugs were registered. Species from the genus *Psallus* were registered as predators only of psyllid genus *Psyllopsis*.

Further investigations are necessary for the preservation of known beneficial predatory true bugs and finding of new ones, potentially usable for biological control on economically significant psyllid species.

## Supplementary Material

XML Treatment for Anthocoris amplicollis

XML Treatment for Anthocoris confusus

XML Treatment for Anthocoris nemoralis

XML Treatment for Anthocoris nemorum

XML Treatment for Orius (Heterorius) majusculus

XML Treatment for Orius (Heterorius) minutus

XML Treatment for Orius (Orius) niger

XML Treatment for Atractotomus mali

XML Treatment for Campylomma verbasci

XML Treatment for Deraeocoris (Deraeocoris) flavilinea

XML Treatment for Deraeocoris (Deraeocoris) ruber

XML Treatment for Deraeocoris (Knightocapsus) lutescens

XML Treatment for Heterocordylus (Heterodactylus) genistae

XML Treatment for Hypseloecus visci

XML Treatment for Malacocoris chlorizans

XML Treatment for Miris striatus

XML Treatment for Orthotylus (Orthotylus) marginalis

XML Treatment for Psallus (Hylopsallus) assimilis

XML Treatment for Psallus (Psallus) flavellus

XML Treatment for Psallus (Phylidea) quercus

XML Treatment for Pseudoloxops coccinea
